# A cytochrome P450 monooxygenase commonly used for negative selection in transgenic plants causes growth anomalies by disrupting brassinosteroid signaling

**DOI:** 10.1186/1471-2229-11-67

**Published:** 2011-04-15

**Authors:** Kasturi Dasgupta, Savita Ganesan, Sindhu Manivasagam, Brian G Ayre

**Affiliations:** 1 University of North Texas, Department of Biological Sciences, 1155 Union Circle #305220, Denton TX 76203-5017, USA; 2 Amyris Biotechnologies, Inc, Emeryville, CA 94608, USA

## Abstract

**Background:**

Cytochrome P450 monooxygenases form a large superfamily of enzymes that catalyze diverse reactions. The *P450*_*SU1 *_gene from the soil bacteria *Streptomyces griseolus *encodes CYP105A1 which acts on various substrates including sulfonylurea herbicides, vitamin D, coumarins, and based on the work presented here, brassinosteroids. *P450*_*SU1 *_is used as a negative-selection marker in plants because CYP105A1 converts the relatively benign sulfonyl urea pro-herbicide R7402 into a highly phytotoxic product. Consistent with its use for negative selection, transgenic Arabidopsis plants were generated with *P450*_*SU1 *_situated between recognition sequences for FLP recombinase from yeast to select for recombinase-mediated excision. However, unexpected and prominent developmental aberrations resembling those described for mutants defective in brassinosteroid signaling were observed in many of the lines.

**Results:**

The phenotypes of the most affected lines included severe stunting, leaf curling, darkened leaves characteristic of anthocyanin accumulation, delayed transition to flowering, low pollen and seed yields, and delayed senescence. Phenotype severity correlated with *P450*_*SU1 *_transcript abundance, but not with transcript abundance of other experimental genes, strongly implicating CYP105A1 as responsible for the defects. Germination and seedling growth of transgenic and control lines in the presence and absence of 24-epibrassinolide indicated that CYP105A1 disrupts brassinosteroid signaling, most likely by inactivating brassinosteroids.

**Conclusions:**

Despite prior use of this gene as a genetic tool, deleterious growth in the absence of R7402 has not been elaborated. We show that this gene can cause aberrant growth by disrupting brassinosteroid signaling and affecting homeostasis.

## Background

Cytochrome P450 monooxygenases (CYPs) form a large superfamily composed of many genes from many organisms. The reactions catalyzed by these enzymes are extremely diverse, but generally involve the transfer of one atom from molecular oxygen to a substrate and reduction of the other atom to form water at the expense of NADPH or NADH [1,2]. CYPs are therefore classified as monooxygenases, but in addition to hydroxylation [3], CYPs can catalyze oxidation [4], dealkylation [5], deamination, dehalogenation and sulfoxide formation [6]. *Arabidopsis thaliana *has 272 predicted CYP genes (246 predicted full-length genes and 26 pseudogene fragments) making it one of the largest gene families in higher plants. The encoded enzymes participate in the anabolism or catabolism of membrane sterols, structural polymers, hormones and many secondary metabolites functioning as pigments, antioxidants and defense compounds. CYP enzymes can also detoxify exogenous molecules such as pesticides and pollutants [1].

CYP enzymes are important regulators of plant growth because they catalyze the synthesis or degradation of several hormones including gibberellins, auxin and brassinosteroids [7]. Brassinosteroids are key hormones involved in cell division and expansion, and are derived from the 30-carbon triterpenoid squalene [8]. CYPs are in this pathway converting squalene to the common membrane sterol campesterol, and also in the brassinosteroid-specific branch pathway that converts campesterol to brassinolide [9]. Specifically, the hydroxylations at C-22 and C-23 have been demonstrated to be catalyzed by CYP90B1, encoded by *DWARF4 *(*DWF4*) [10-12] and CYP90A1 [13], encoded by *CONSTITUTIVE PHOTOMORPHOGENESIS AND DWARFISM *(*CPD*), respectively, by genetic, biochemical, and molecular analyses in Arabidopsis. Auxins also regulate many aspects of growth and development, and CYP79B2 and CYP83B1 participate in tryptophan-dependent indole acetic acid (IAA) synthesis [7,14]. Gibberellins (GAs) are tetracyclic diterpenoid compounds which play important roles in germination, stem elongation and reproductive development [15]. GAs are synthesized by a pathway involving three enzyme classes spanning different subcellular compartments [16]. The steps of the pathway from ent-kaurene to GA_12 _are catalyzed by CYP88A and CYP701A family members, and CYP714D1 participates in GA deactivation [16,17].

CYP enzymes are also involved in detoxifying exogenous molecules. This is best studied in animal systems where CYPs have significant pharmaceutical impact, but action against xenobiotics is also observed in bacteria, fungi and plants [2]. In plants, commonly used herbicides such as prosulfuron, diclofop and chlortoluron can be detoxified by CYPs. In weeds, herbicide resistance can arise from elevated CYP activity, which is particularly problematic because it can increase resistance to a broad class of related molecules [18]. In the case of the phenylurea herbicide, chlortoluron, CYP-mediated detoxification is achieved either by hydroxylation of the ring-methyl or by di-N-demethylation [1,19]. In addition, CYP genes from other organisms have been used for engineering herbicide resistance in plants, as well as for developing new herbicides in conjunction with cognate antidote genes conferring resistance. Understanding and manipulating the association between herbicides and herbicide-resistance genes is therefore a prominent goal for agricultural biotechnology [18].

The *P450*_*SU1 *_gene from the soil bacteria *Streptomyces griseolus *encodes an inducible cytochrome P450, CYP105A1, capable of metabolizing sulfonylurea herbicides via dealkylation [20]. However, the activity of CYP105A1 also results in the metabolism of the sulfonylurea pro-herbicide 2-methylethyl-2, 3-dihydro-N-[(4, 6-dimethoxypyrimidin-2-yl) aminocarbonyl]-1, 2-benzoisothiazole-7-sulfonamide-1, 1-dioxide (R7402) to a highly phytotoxic metabolite, such that plants expressing *P450*_*SU1 *_are killed by R7402 treatment at levels that are benign to plants without *P450*_*SU1 *_expression. This has allowed *P450*_*SU1 *_to be used in conjunction with R7402 as a negative-selection marker to select for plants that lack *P450*_*SU1 *_as a transgene [20]. Negative selection markers like *P450*_*SU1 *_are useful in experiments where selecting for the loss of genes linked to the marker is desired. For example *P450*_*SU1 *_has been used in *Ac*/*Ds *transposon-mediated mutagenesis screens to select for progeny in which the *Ac *transposase gene had segregated away from the *Ds *element, thereby ensuring that the location of the *Ds *element was stable after the initial *Ac*-mediated transposition event [21]. In addition, negative-selection markers are commonly used in combination with site-specific recombinases and serve as a screening tool for selecting the desired recombinase-mediated excision event. For example, to demonstrate the utility of the *P450*_*SU1*_/R7402 negative-selection system for crop plants and biotechnology, it was used to select transgenic barley in which the transgene of interest was retained, but the gene encoding antibiotic resistance was linked to *P450*_*SU1 *_and lost by recombinase-mediated excision [22].

The work reported in this study initiated as an effort to select for plants that had lost a cDNA sequence encoding a Suc/H^+ ^symporter necessary for efficient Suc transport through the phloem [23]. The cDNA for *AtSUC2 *and *P450*_*SU1 *_were placed between target sequences for *Saccharomyces cereviseae *FLP recombinase, with the intention of using R7402 to select for efficient FLP-mediated excision of the cassette. However transgenic Arabidopsis plants transformed with this construct displayed a range of aberrant growth phenotypes, with more extreme lines exhibiting dwarfing, rosettes with a distinctive spiral-growth habit, delayed transition to flowering, low pollen yields and fecundity, and delayed senescence. These phenotypes have not been described in plants with altered *AtSUC2 *expression but resemble those described for plants with disrupted brassinosteroid signaling [11,13,24]. We describe experiments correlating the severity of the phenotypes with *P450*_*SU1 *_expression levels and not *AtSUC2 *expression levels, and report on further experiments indicating that CYP105A1 from *S. griseolus *disrupts brassinosteroid homeostasis in these transgenic plants.

## Results

### Arabidopsis lines overexpressing *P450*_*SU1 *_show abnormal growth

**Figure 1:**
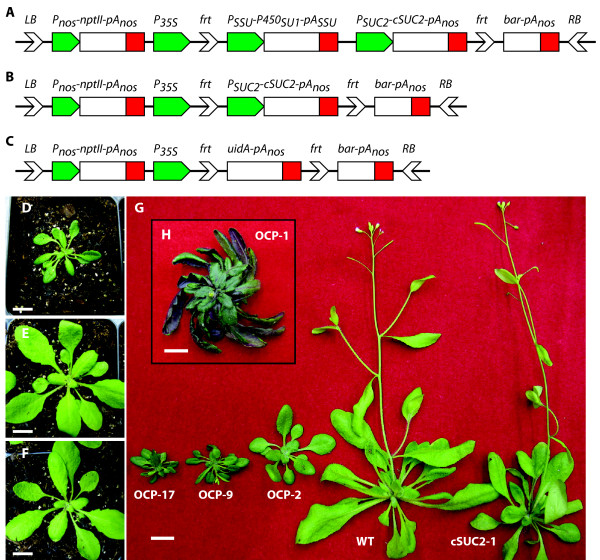
**T-DNA cassettes used in this study and representative Arabidopsis plants displaying a range of aberrant and normal growth patterns**. Schematic representation of T-DNA cassettes in **(A) **pART-P450-cSUC2-BAR, **(B) **pART-cSUC2-BAR, and **(C) **pART-uidA-BAR. LB: T-DNA left border; RB: T-DNA right border; *P*_*nos*_*-nptII-pA*_*nos*_: nopaline synthase promoter - neomycin phosphotransferase cDNA - nopaline synthase poly-adenylation signal; *P*_*35S*_: Cauliflower Mosaic Virus 35S promoter; *frt: *FLP recombinase recognition target sites; *P*_*SSU*_*-P450*_*SU1*_*-pA*_*SSU*_: Rubisco small subunit promoter - *P450*_*SU1 *_gene encoding CYP105A1 cytochrome P450 monooxygenase - Rubisco small subunit poly-adenylation signal; *P*_*SUC2*_*-cSUC2-pA*_*nos*_: 2 kb of *AtSUC2 *promoter - excisable cDNA of *AtSUC2 *- nopaline synthase poly-adenylation signal; *bar-pA*_*nos*_: Basta (glufosinate ammonium) resistance cDNA - nopaline synthase poly-adenylation signal. Representative 21-day old rosettes of **(D) **transgenic line OCP-1 (Overexpressing Cytochrome *P450*_*SU1*_) harboring pART-P450-cSUC2-BAR and displaying a severe phenotype, **(E) **transgenic line cSUC2-1 harboring pART-cSUC2-BAR, and **(F) **wild type Arabidopsis. **(G) **Representative 35-day old OCP-17, OCP-9 (both displaying severe phenotypes), OCP-2 (displaying a moderate phenotype), wild type, and cSUC2-1, as indicated. **(H, inset) **Representative 50-day old OCP-1 plant showing anthocyanin accumulation and 'twirled' rosette. Scale bar in D - H is 1 cm.

The plasmid pART-P450-cSUC2-BAR (Figure 1A) was used to create transgenic plants with an excisable *AtSUC2 *cDNA (*cSUC2*) adjacent to the negative selection marker *P450*_*SU1*_. *AtSUC2 *encodes the predominant Suc/H^+ ^symporter required for efficient phloem loading and transport, and plants harboring a homozygous mutation are severely debilitated [23,25]. Transgenic plants with an excisable *cSUC2 *cassette would be a valuable research tool and alleviate some of the difficulties associated with null mutants. The negative-selection gene *P450*_*SU1 *_was incorporated into the excisable cassette as a marker for effective excision. *P450*_*SU1 *_encodes CYP105A1, a CYP from *Streptomyces griseolus *which converts the relatively benign pro-herbicide R7402 into a highly phytotoxic product. In the presence of R7402, whole plants or tissues expressing *P450*_*SU1 *_die while those having lost the sequences retain viability [20]. Similarly, plasmids pART-cSUC2-BAR and pART-uidA-BAR (Figure 1B, C) were used to create transgenic plants used as controls in the experiments.

Growth aberrations on sterile media during selection on kanamycin and in potting mix were noted among a large proportion of independent T1 seedlings harboring pART-P450-cSUC2-BAR (referred to as OCP lines; Overexpressing Cytochrome *P450*_*SU1*_). In plants displaying the most severe phenotype, these aberrations included severe stunting, darker green and purplish leaves characteristic of anthocyanin accumulation, thicker leaves in the abaxial/adaxial orientation, delayed flowering, shortened inflorescence internodes, reduced apical dominance (Figure 1D-G), and numerous unexpanded siliques with no or very few seeds. In addition, plants with the most severe phenotype demonstrated counter-clockwise leaf curling that gave rosettes a distinctive 'twirled' appearance (Figure 1H). Similar phenotypes were not observed in T1 plants (n > 20) harboring pART-cSUC2-BAR or pART-uidA-BAR, or in any WT plants.

The two antibiotic genes, *nptII *and *bar*, are common markers that are present in all three T-DNA sequences: they are unlikely to be responsible for the growth abnormalities observed in plants transformed with pART-P450-cSUC2-BAR. Reduced or ectopic expression of genes encoding Suc/H^+ ^symporters can disrupt patterns of carbon partitioning and cause growth anomalies, such as stunting, anthocyanin accumulation, and low seed yield [26-28]. However, growth aberrations were not observed among pART-cSUC2-BAR plants (referred as cSUC2 lines), and altered carbon partitioning does not account for the full spectrum of phenotypes observed among pART-P450-cSUC2-BAR plants. *P450*_*SU1 *_has been used as a negative-selection marker in tobacco, Arabidopsis and barley [20-22]. In barley, "striking morphological differences" were observed in transgenic plants compared to non-transgenic plants [22]. However, elaboration of those differences was not provided, and no morphological changes are described for Arabidopsis or tobacco.

### Transcript levels of *P450*_*SU1 *_correlate with the aberrant phenotype

**Figure 2:**
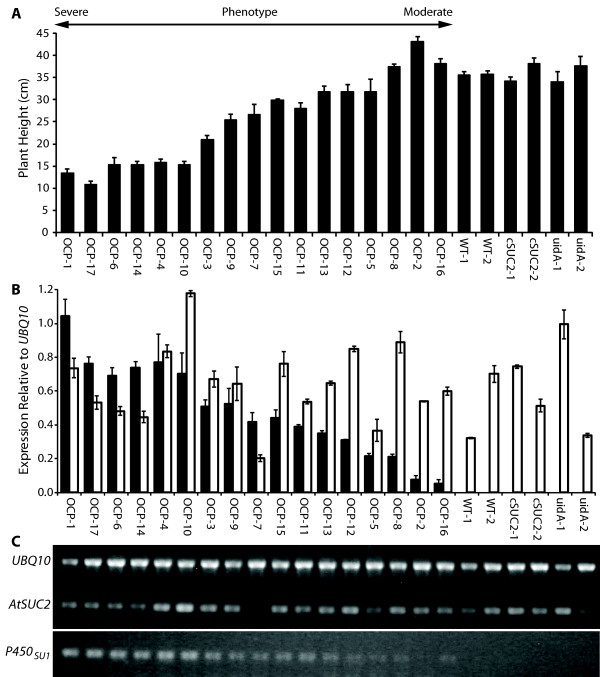
**Relationships between aberrant growths, represented as plant height, and *AtSUC2 *and *P450***_***SU1***_** transcript abundance**. **(A) **OCP, WT, cSUC2, and uidA lines arranged by phenotype severity, with plant height of the indicated lines at full maturity (*i.e*., senescent and ready for seed harvesting), n = 6, variation is expressed as standard deviation. **(B) **Semi-quantitative RT-PCR of *P450*_*SU1 *_(black bars) and *AtSUC2 *(white bars) transcript levels relative to *UBQ10 *transcript, encoding ubiquitin, n = 3, variation is expressed as standard deviation. **(C) **Representative gel used to calculate transcript abundance. See Materials and Methods for details.

The extent of the phenotype varied among OCP lines independently transformed with pART-P450-cSUC2-BAR and suggested a correlation with expression of one of the transgene: most likely *P450*_*SU1 *_but possibly *AtSUC2*. *P450*_*SU1 *_and *AtSUC2 *transcript levels were analyzed relative to *UBQ10 *transcripts (encoding ubiquitin) by semi-quantitative RT-PCR in 17 OCP lines, as well as in WT and cSUC2 lines, and those transformed with pART-uidA-BAR (uidA lines) (Figure 2). In Figure 2, the OCP lines were ranked by height for severity of phenotype in 50-day old plants and there is a strong correlation between *P450*_*SU1 *_transcript level and phenotype: Lines with the most severe phenotype had the highest levels of *P450*_*SU1 *_transcript while those with intermediate and no phenotype had lesser and no transcript, respectively (Figure 2). Conversely, *AtSUC2 *and *cSUC2 *transcript levels (the oligonucleotides used for qRT-PCR detect transcript from both) showed variation among lines with no obvious correlation to phenotype. These findings strongly suggest that expression levels of *P450*_*SU1*_, and thus levels of CYP105A1 protein, interfere with plant growth and development.

### Over expression of *P450*_*SU1 *_affects vegetative and reproductive growth

**Figure 3:**
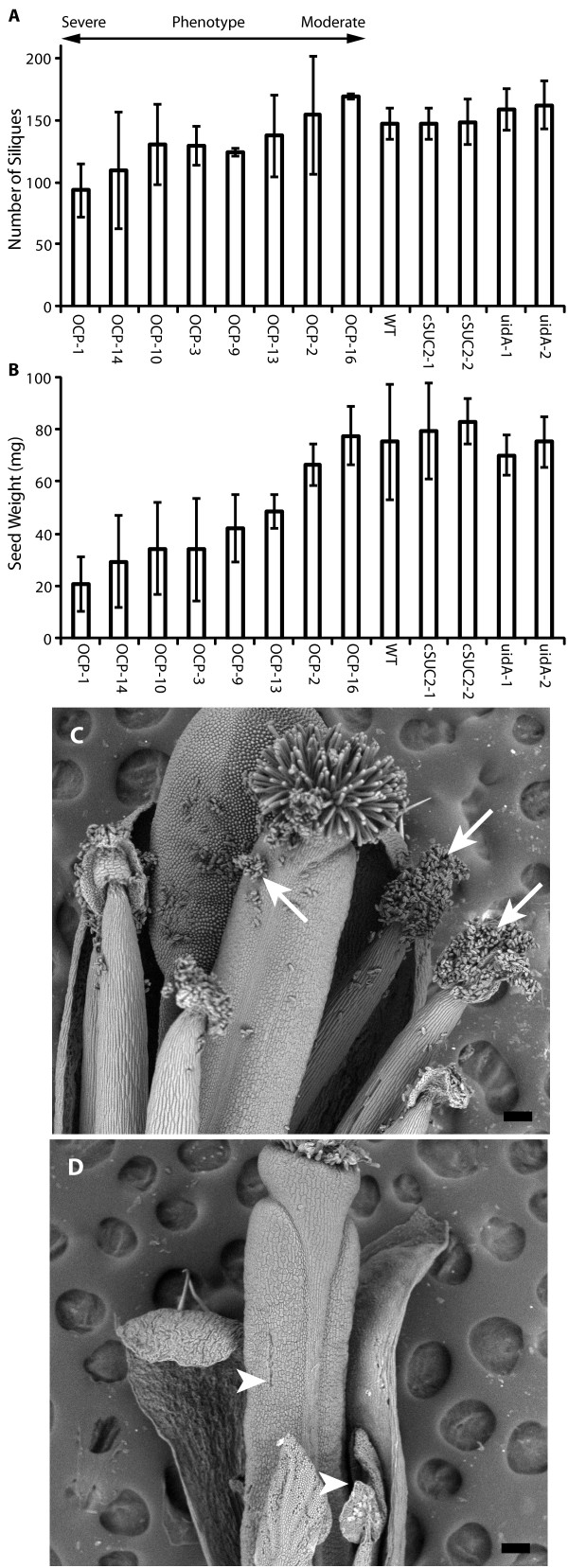
**Fecundity analyses of representative OCP lines relative to WT, cSUC2 and uidA control lines**. **(A) **Number of siliques per plant on the indicated lines at maturity. **(B) **Seed yield per plant harvested from indicated lines. OCP lines are arranged by phenotype severity and variation is expressed as standard deviation, n = 10. Scanning electron micrographs of a **(C) **WT flower showing copious pollen on anthers and carpels (arrows) and **(D) **OCP-1 flower with a dearth of pollen (arrowheads). Flowers in (C) and (D) are the same age with respect to opening (anthesis), some petals and sepals were removed to view the internal organs, scale bar is 100 μm.

**Figure 4:**
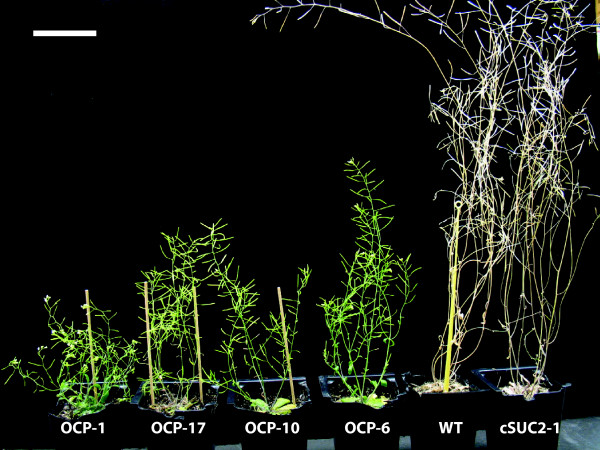
**Delayed senescence in OCP lines relative to WT and cSUC2 lines**. 60-day old representative plants of the indicated lines. Note the shortened internodes and lack of senescence among the OCP plants; OCP-1 still has active blooms. Scale bar is 5 cm.

Having established a correlation between *P450*_*SU1 *_expression and phenotype, a more detailed analysis of OCP growth and development was conducted. Representative lines demonstrating severe, intermediate, and mild phenotypes were analyzed relative to WT, cSUC2 and uidA lines as controls. As shown in Table 1, the reproductive phase of the OCP lines was significantly delayed: Under long-day conditions, WT, cSUC2 and uidA lines had visible floral organs within 24-26 days while *P450*_*SU1 *_expression associated with delayed transition to flowering (Table 1). Plants overexpressing *P450*_*SU1 *_also had fewer siliques and individual siliques had fewer seeds, resulting in an overall lower seed yield (Figure 3A, B). To gain insight into why fecundity in OCP lines was compromised, scanning electron microscopy was used to analyze flower development. Most conspicuous was the near absence of pollen in severe OCP lines (Figure 3C, D), which may account partially or entirely for the reduced seed yield. Additionally, OCP lines had delayed senescence: 60-day old OCP plants had green leaves and siliques while WT and cSUC2 lines had completely senesced (Figure 4). Seed size was not affected but germination varied among the OCP lines whereas it was consistently high among WT, cSUC2, and uidA lines (data not shown).

**Table 1:** Effect of *P450*_*SU*__*1 *_on flowering time in OCP lines

Plant line	Days to flower	Total number of leaves
OCP-1	51.2 ± 1.1^a^	42.5 ± 1.8^a^
OCP-10	50.2 ± 1.3^a^	50.2 ± 0.8^a^
OCP-3	42.6 ± 2.3^a^	38.1 ± 1.9^a^
OCP-9	32.7 ± 3.5^a^	25.7 ± 2.6^a^
OCP-13	34.7 ± 3.2^a^	27.0 ± 2.2^a^
OCP-2	28.5 ± 1.8^a^	19.7 ± 3.4
WT	24.5 ± 0.8	12.3 ± 0.4
cSUC2-1	25.0 ± 2.8	14.8 ± 2.1
uidA-1	24.8 ± 2.5	13.5 ± 2.5

Data represents mean values ± standard deviation of 12 plants from different OCP and control lines.

^a ^Student's T-test, p < 0.05, relative to wild type (WT).

### Overexpression of *P450*_*SU1 *_impacts brassinosteroid homeostasis

The morphological and developmental anomalies observed among OCP lines are characteristic of plants defective in brassinosteroid (BR) synthesis and signaling. Plants defective in BR synthesis and signaling display characteristic phenotypes that include severe stunting, darker color from anthocyanin accumulation, epinastic round leaves, delayed flowering, late senescence, reduced male fertility, and compromised germination [13,24,29-31]. Seedlings deficient in BR signaling also undergo abnormal skotomorphogenesis [29]. Unlike the elongated hypocotyls, closed cotyledons and prominent apical hooks of WT Arabidopsis seedlings germinated and grown in the dark, BR-deficient seedlings exhibit short and thickened hypocotyls, open and expanded cotyledons, and the emergence of true leaves characteristic of the de-etiolation that occurs during photomorphogenesis [32,33]. Exogenous BR can stimulate cell division and expansion and rescue biosynthetic mutants. In WT plants, exogenous BR can cause supraoptimal effects and result in abnormal development from chaotic growth [13].

**Figure 5:**
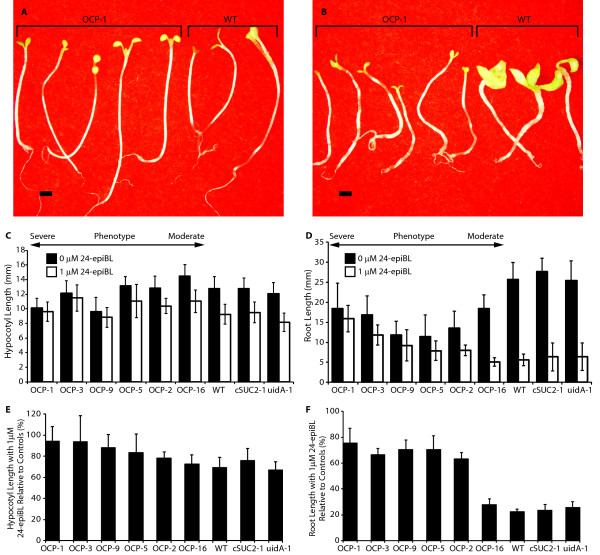
**Expression of *P450***_***SU1***_** affects hypocotyl and root growth in the dark in the presence and absence of exogenous 24-epibrassinolide**. Images of dark-grown 5-day old seedlings from OCP-1 and wild type in the **(A) **absence and **(B) **presence of exogenous 1 μM 24-epiBL. Scale bar is 1 mm. **(C) **Hypocotyl length and **(D) **root length in the absence (black bars) and presence (white bars) of 1 μM 24-epiBL. **(E) **Hypocotyl length and **(F) **root length in the presence of 1 μM 24-epiBL relative to sibling plants grown in the absence of exogenous hormone. OCP lines are arranged by phenotype severity, and variation is expressed as SD; n = 12 sibling plants.

To test if *P450*_*SU1 *_expression in the OCP lines affects BR signaling, the impact of exogenous 24-epibrassinolide (24-epiBL) on skotomorphogenesis was analyzed in dark grown seedlings. In the absence of 24-epiBL, severe OCP lines showed moderate reductions in hypocotyl elongation relative to less severe lines and controls (Figure 5A, C). In the presence of supraoptimal 1 μM 24-epiBL, importantly, severe OCP lines showed no significant alteration in growth while WT and other control seedlings displayed substantial morphological disruptions including chaotic growth in hypocotyls and cotyledons (compare Figure 5A and 5B) and generally shorter hypocotyls (Figure 5E).

BR levels are also known to impact root development. Mutants deficient in BR or BR signaling have shorter roots than WT and in the presence of supraoptimal exogenous BR, root development can be severely impaired [34-36]. Root growth was measured in OCP and WT lines on vertically-oriented sterile media. In the absence of exogenous 24-epiBL, OCP lines had shorter roots than WT but this did not correlate strongly with the severity of the above-ground phenotype (Figure 5D). In the presence of 1 μM 24-epiBL, the length of WT roots was reduced to 22% of roots grown in the absence of 24-epiBL, whereas roots of the most severe OCP lines were reduced to only 65% to 75% relative to those grown without exogenous 24-epiBL (Figure 5D, F). These findings that exogenous 24-epiBL severely affects WT root and aerial growth, but has little impact on the most severe OCP lines, combined with a growth pattern that phenocopies BR deficient mutants (described above), strongly suggests that the CYP105A1 enzyme encoded by the *P450*_*SU1 *_gene is affecting BR homeostasis directly or indirectly.

### Overexpression of *P450*_*SU1 *_does not impact gibberellin or auxin mediated growth characteristics

**Figure 6:**
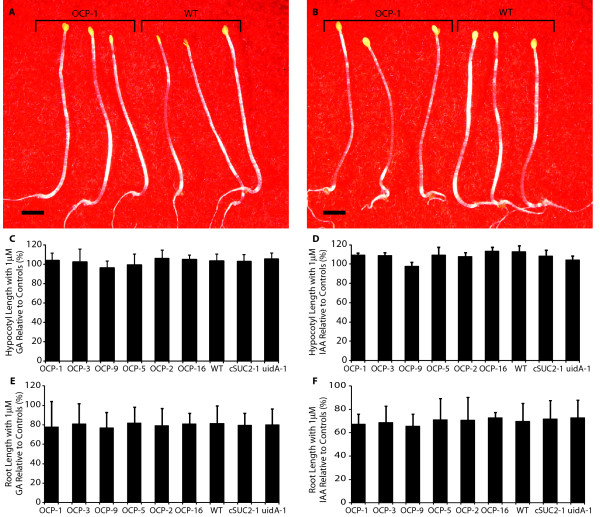
**Expression of *P450***_***SU1***_** does not influence the impact of GA**_**3 **_**or IAA on hypocotyl and root growth**. Images of dark-grown 5-day old seedlings from OCP-1 and wild type in **(A) **the presence of 1 μM GA_3_, and **(B) **the presence of 1 μM IAA. Scale bar is 1 mm. **(C, D) **Hypocotyl length and **(E, F) **root length in the presence of 1 μM GA_3 _(C, E) and 1 μM IAA (D, F) relative to sibling plants grown in the absence of exogenous hormone. OCP lines are arranged by phenotype severity, and variation is expressed as SD; n = 12 sibling plants.

Gibberellin and auxin metabolism are also impacted by CYP activity, and hypocotyl- and root-growth experiments were conducted to test if CYP105A1 visually affects growth responses to these hormones. Exogenous application of GA_3 _or IAA is known to modestly increase hypocotyl length of etiolated seedlings [37-39]. This was observed in wild type and control plants, but the effect was identical among even the most severe OCP lines (Figure 6A-D; the slight decrease in observed in OCP9 is not statistically significant). Conversely, exogenous GA_3 _or IAA treatment is known to result in decreased root elongation in etiolated seedlings [14,37,40]. In our experiments with 1 μM of either hormone, OCP and control lines showed identical extents of reduced root elongation (Figure 6E, F). These results show that *P450*_*SU1 *_expression does not mitigate the influence of exogenous GA_3 _or IAA (Figure 6) as it did for exogenous 24-epiBL (Figure 5), and argues that the CYP105A1 enzyme impacts BR homeostasis, but not that of IAA or GA_3_.

## Discussion

This study initiated as an effort to create a vector system in which a cDNA sequence of interest could be excised upon delivery or activation of a site-specific recombinase. It was designed with dual selection for recombination. After FLP-mediated recombination at the *frt *sites, the positive selection marker *bar *(also *pat*; phosphinonothricin aminotransferase) was to be activated by being placed adjacent to a CaMV 35S promoter [41] and the negative selection marker *P450*_*SU1 *_was to be inactivated by being excised from the genome along with the cDNA of interest (cDNA encoding the AtSUC2 Suc/H^+ ^symporter in this specific case). Independent transgenic lines harboring this construct displayed a range of phenotypes with the most severe lines resembling plants with disrupted BR synthesis or perception [9]. This included stunted rosettes and inflorescences with short internodes and reduced apical dominance, thicker leaves with dark coloration characteristic of anthocyanin accumulation, leaf curling that gave rosettes a distinctive twirled appearance (Figure 1), reduced male fertility and seed yields (Figure 3 and Table 1), and delayed senescence (Figure 4). The severity of these characteristics showed a high correlation with *P450*_*SU1 *_expression levels (Figure 2), and on sterile media these lines showed the least response to supraoptimal levels of 24-epiBL (Figure 5). As controls, plants transformed with T-DNA that retained the *AtSUC2 *cDNA but had *P450*_*SU1 *_deleted were phenotypically normal, as were plants lacking both *AtSUC2 *cDNA and *P450*_*SU1 *_and instead expressing *uidA *encoding β-glucuronidase. The combined results of (1) the close correlation between *P450*_*SU1 *_expression and a phenotype resembling a deficiency in BR synthesis or perception, (2) *P450*_*SU1 *_expression mitigating the effects of exogenous 24-epiBL, and (3) the process of eliminating other candidate genes indicate that the CYP105A1 enzyme is acting on exogenous BR and affects endogenous BR by altering BR homoeostasis. A T-DNA construct harboring only *P450*_*SU1 *_was not tested. Expression of *P450*_*SU1 *_did not modify the growth of etiolated seedlings in the presence of IAA or GA_3_, indicating that it does not act on these hormones (Figure 6).

*P450*_*SU1 *_and the encoded enzyme CYP105A1 were originally identified from the soil bacterium *Streptomyces griseolus *as being able to degrade sulfonylurea herbicides [20]. In transgenic plants, CYP105A1 converted the relatively benign compound R7402 into a highly phytotoxic herbicide and could thus be used for negative selection: plants or individual tissues expressing *P450*_*SU1 *_were ablated by R7402 application, while plants or tissues not expressing the gene were spared [20]. *P450*_*SU1 *_was used previously in several studies, but we are aware of only one were growth aberrations in the absence of R7042 were noted. Specifically, Koprek and colleagues [22] compared the efficacy of *P450*_*SU1 *_and the *codA *gene, which converts non-toxic 5-fluorocytosine to toxic 5-fluorouracil [42], as negative-selection tools in transgenic barley. The abstract of [22] notes growth anomalies with *P450*_*SU1 *_but did not elaborate, and the authors concluded that despite these anomalies, *P450*_*SU1 *_along with R7042 was suitable for negative selection among plants grown in soil. Based on our findings, the growth anomalies reported in barley [22] are likely the result of perturbed brassinosteroid signaling.

There are several explanations as to why a link between *P450*_*SU1 *_and growth aberrations from perturbed brassinosteroid signaling have not been reported. First, the system is used for negative selection in conjunction with R7402 and production of the phytotoxic byproduct results in rapid death of plants or tissues. Therefore, the effects of *P450*_*SU1 *_in the absence of R7402 are mild compared to the effects in the presence of R7402. Second, since the system is used for negative selection, most attention has focused on characteristics of plants or tissues after loss of the gene by segregation, transposition, or recombination [43]. Third, in the unique vector system used here, a strong CaMV 35S promoter was placed upstream of a strong Rubisco promoter (Figure 1A), and this combination may result in expression levels higher than those obtained in studies where growth anomalies were not reported. This is supported by the strong correlation between transcript abundance and phenotype severity. Lines with moderate to low *P450*_*SU1 *_transcript levels displayed moderate to mild symptomology in the absence of R7402, but were still highly sensitive to R7402 and suitable for negative selection (data not shown). In addition, CYP105A1 as used here is targeted to plastids [20] and expression from a dual promoter system may overwhelm plastid targeting and result in more enzyme mislocalized to the cytosol for acting on BRs. Potential mislocalization of plastid-targeted CYP105A1 was previously reported [20].

The dual promoters may also explain discrepancies between the phenotypes of our most severe lines and mutants defective in BR synthesis. For example, in the *CPD *mutant which is disrupted in BR synthesis, dark-grown seedlings show photomorphogenesis and have short, thickened hypocotyls [13] but our most severe OCP line showed normal skotomorphogenesis and differed only moderately from WT. The Rubisco small subunit promoter is light-activated, and in dark-grown seedlings expression would have been minimal. Under these conditions, *P450*_*SU1 *_expression from the more distal CaMV 35S promoter alone may have been insufficient to cause a more severe phenotype. However, in the presence of 24-epiBL, OCP seedlings likely had sufficient *P450*_*SU1 *_expression to bring brassinosteroid levels into a range that allowed relatively normal development.

As described above, CYP105A1 metabolizes sulfonylurea herbicides by dealkylation. Sulfonylurea herbicides are agricultural soil additives, and the natural target and substrate specificity of CYP105A1 is not known. In transgenic plants, CYP105A1 disrupts brassinosteroid homeostasis to give a phenotype, but the full range of potential substrates and the extent to which their levels are altered is not known. Work by others has shown that CYP105A1 can hydroxylate vitamin D2 and D3 at multiple positions [44] and can catalyze the conversion of 7-ethoxycoumarin to 7-hydroxycoumarin by O-dealkylation [3]. Detoxification of sulfonylurea herbicides and N-dealkylation of the pro-herbicide R7402 to produce a toxic metabolite are additional activities [20], and collectively, these reactions suggest that CYP105A1 substrate selection and mode of action may be quite broad, but does not extend to IAA or GA_3_.

It is now apparent that the development of herbicide resistance in several weeds is the result of enhanced detoxification associated with elevated levels of CYP activity. Weeds with enhanced CYP-mediated detoxification can be difficult to control because resistance can develop against multiple, unrelated classes of herbicide [18,45]. However, in the limited species that have been subjected to analysis, there is a fitness cost associated with elevated CYP levels: In the absence of the selective pressure imparted by the herbicide, herbicide-resistant varieties of *Lolium rigidum *showed up to 30% reduced vitality relative to their herbicide-sensitive counterparts [46]. The development of CYPs from both plant and non-plant origins for engineering herbicide resistance in biotechnology has garnered substantial interest. However, the reduced vitality of plants that have naturally developed resistance and the undesired effects on plant-hormone homeostasis observed here with overexpression of *P450*_*SU1 *_highlight some of the potential deleterious outcomes that will need to be addressed for successful exploitation of this promising area.

## Conclusions

The *P450*_*SU1 *_gene from *Streptomyces griseolus *has been used as a negative-selection marker in conjunction with the pro-herbicide R7402 since plants expressing the gene are killed by R7402 while those not expressing it retain viability. However, in the absence of R7402, plants with high *P450*_*SU1 *_expression show aberrant growth characteristic of defects in brassinosteroid synthesis and perception. When exposed to supraoptimal exogenous brassinosteroids, the growth habit of these plants is relatively normal compared to wild type. Together, these results indicate that both endogenous and exogenous brassinosteroids are a target of the *P450*_*SU1 *_encoded CYP105A1 monooxygenase.

## Methods

### Plasmid construction

Unless stated otherwise, plasmids were created by standard protocols [47], enzymes were obtained from New England Biolabs (Beverly, MA) and correct constructs were verified by sequencing (SeqWright, Houston TX). The starting material for the plasmids used in this study (Figure 1) was pFLP-SWITCH [41]. The *Bar *gene encoding resistance to glufosinate ammonium herbicide was amplified from pGPTV-BAR [48] using primers BARKpn3 (5'-AGTAAGGTACCTCATCAGATTTCGGTGACG-3') and BARHind5 (5'-TTACTAAGCTTAACAATGAGCCCAGAACGACG-3'). The amplified product was ligated to itself and used as the template for PCR with BARKpnmut3 (5'-ACGGGGCGGAACCGGCAGGCTGAAG-3') and BARKpnmut5 (5'-CCGGTCCTGCCCGTCACCGAAATC-3'), which mutated an internal *Kpn*I site without altering the encoded amino acid sequence. This product was ligated to itself and used as the template in a final round of PCR using BARKpn3 and BARHind5. This mutagenized *Bar *PCR product was digested with restriction enzymes *Hind*III and *Kpn*I and ligated into the same sites of pFLP-SWITCH to create pFLP-SWITCH-BAR. pGEM-uidA-BAR was created by inserting the *Not*I cassette of pFLP-SWITCH-BAR into the *Not*I site of a pGEM T-easy (Promega, Wisconsin, USA) derivative in which the *Sac*I site in the multiple cloning site was removed by digesting with *Sac*I, using T4 DNA Polymerase to make blunt ends, and religating the plasmid backbone. The *uidA *gene in pGEM-uidA-BAR was replaced with *AtSUC2 *cDNA (*cSUC2*) from pGEM-SUC2p::cSUC2 [23] using *BamH*I and *Sac*I to create pGEM-cSUC2-BAR. Plasmid pSSU-SU11 with a *P450*_*SU1 *_gene cassette consisting of a promoter from the small subunit of Rubisco, a chloroplast targeting sequence fused to the *P450*_*SU1 *_open reading frame and a polyadenylation signal from Rubisco was obtained from Daniel O'Keefe [20]. A fragment of this cassette was PCR-amplified with the oligonucleotide PspOMImutR2496 (5'-AATAACGGGGCCCCCCGCGATGTC-3') to mutate the internal *Not*I restriction site to a *PspOM*I restriction site and the oligonucleotide *Bgl*IImutF7518 (5'-CATGATTACGAATTCTAGATCTTCTCTGC-3') to introduce a *Bgl*II site at the 5' end of the cassette. The PCR product was digested with *EcoR*I and *PspOM*I and ligated into *EcoR*I and *Not*I digested pSSU-SU11 to create pUC118-P450mut. The *P450*_*SU1 *_cassette was then excised with *BamH*I and *Bgl*II and ligated into *BamH*I digested pGEM-cSUC2-BAR to create pGEM-P450-cSUC2-BAR. The orientation of the *P450*_*SU1 *_cassette recreated the *BamH*I restriction site between the *P450*_*SU1 *_gene and *cSUC2*. pGEM-P450-cSUC2-BAR was digested with *Not*I and the P450-cSUC2-BAR fragment ligated into the *Not*I site of the binary vector pART27 [49] generating pART-P450-cSUC2-BAR. Similarly, pGEM-cSUC2-BAR and pGEM-uidA-BAR were digested with *Not*I to introduce the cSUC2-BAR and uidA-BAR cassettes, respectively, into pART27 to generate pART-cSUC2-BAR and pART-uidA-BAR. In all binary vectors, the orientations of the genes in the cassettes were the same as the pART27 *nptII *gene.

### Plant Material and Growth Conditions

Seeds were stratified at 4°C for 48 hours prior to germination, and plants were grown in a Percival AR95L chamber (Percival Scientific, Perry, IA) with 14 h light/10 h dark at 21°C. Plants with the *Atsuc2-4 *allele (SALK_038124) have a T-DNA insertion in *AtSUC2 (At1g22710*) [23]. Heterozygous plants (*AtSUC2/Atsuc2-4*) were transformed [50] with pART-P450-cSUC2-BAR, pART-cSUC2-BAR, and pART-uidA-BAR, and T1 seedlings selected on Murashige and Skoog basal medium with Gamborg vitamins (Phytotechnology Laboratories, Shawnee Mission, KS) containing 100 mg L^-1 ^of kanamycin for seven days before transferring to MetroMix 360 potting media (Sun Gro Horticulture, Vancouver, Canada). Rosettes were digitally photographed 21 days post-germination, just before WT plants transitioned to flowering, such that all aerial growth was represented in rosette area. For root and hypocotyl growth analysis, seeds were germinated on vertically-oriented MS plates, supplemented with 100 mg L^-1 ^kanamycin and 1 μM 24-epibrassinolide (24-epiBL), gibberellic acid (GA_3_) (both from PhytoTechnology Laboratories), or indole acetic acid (IAA) (Sigma) as indicated and seedlings were analyzed after 7 days. For experiments with dark-grown seedlings, stratified seeds on sterile medium were exposed to light for three hours to induce germination and then covered with aluminum foil for five days. Digitally-photographed plants were analyzed using Image J [51]. To assess pollen abundance, flowers of 40-day old plants were imaged with a Hitashi TM-1000 scanning electron microscope after removing some of the sepals and petals.

### Transcript analysis

Total RNA was isolated from rosette leaves of 21-day old plants using Trizol (Invitrogen Carlsbad, CA) according to the manufacturer's instructions and treated with RNase-free DNaseI (Invitrogen). 500 ng RNA from each plant was reverse transcribed with 50 μM oligo(dT) and SuperScript III reverse transcriptase (Invitrogen) according to the manufacturer's instructions. For semiquantitative PCR, 1 μL of cDNA was amplified in the presence of 250 μM dNTP and 500 nM each forward and reverse primer in 25 μL reactions with RedTaq Genomic DNA Polymerase (Sigma-Aldrich, St. Louis, MO). Cycling parameters were 94°C for 10 s, 60°C for 15 s, and 72°C for 50 s. 25, 30, and 35 cycles (in separate tubes) were tested for increasing band intensities, and three replicates of 30 cycles and 35 cycles were used to quantify band intensity with ImageJ [51] by resolving 5 to 10 μL on 1.5% agarose gels. Oligonucleotides amplifying *AtSUC2 *sequences downstream of the T-DNA insert were AtSUC2Ex3Ex4F (5'-TAGCCATTGTCGTCCCTCAGATG-3'; spans the junction between exons 3 and 4) and SUC2-3-ORF (5'-ATGAAATCCCATAGTAGCTTTGAAGG-3'). Oligonucleotides specific to *P450*_*SU1 *_were RT5P450 (5'-GTGCAGTCCACGGACGCGCAGAG-3') and P4501RT3 (5'-CGATGGCGAGGTAGCGGAGCAGTTC-3'). Transcript abundance was standardized to *UBQ10 *(encoding ubiquitin), using oligonucleotides UBQ1 (5'-GATCTTTGCCGGAAAACAATTGGAGGATGGT-3') and UBQ2 (5'-CGACTTGTCATTAGAAAGAAAGAGATAACAGG-3') [52].

## Authors' contributions

KD and SG made the plasmids, created the transgenic plants, and with SM, analyzed the plants. KD and BGA designed the experiments. KD and BGA wrote the manuscript. All authors read and approved the final manuscript.
